# Headache, anosmia, ageusia and other neurological symptoms in COVID-19: a cross-sectional study

**DOI:** 10.1186/s10194-021-01367-8

**Published:** 2022-01-03

**Authors:** Pedro Augusto Sampaio Rocha-Filho, Pedro Mota Albuquerque, Larissa Clementino Leite Sá Carvalho, Mylana Dandara Pereira Gama, João Eudes Magalhães

**Affiliations:** 1 Division of Neuropsychiatry, Centro de Ciências Médicas, Universidade Federal de Pernambuco (UFPE), Av. da Engenharia, 531-611, Recife, PE 50730-120 Brazil; 2 Hospital Universitario Oswaldo Cruz, Universidade de Pernambuco, Tv. Jackson Pollock - Santo Amaro, Recife, PE 52171-011 Brazil

**Keywords:** Neurologic manifestations, COVID-19, SARS-CoV-2, Headache, Anosmia, Ageusia

## Abstract

**Background:**

Neurological symptoms are frequent among patients with COVID-19. Little is known regarding the repercussions of neurological symptoms for patients and how these symptoms are related to one another.

**Objectives:**

To determine whether there is an association between the neurological symptoms in patients with COVID-19, and to characterize the headache.

**Method:**

This was a cross-sectional study. All hospital inpatients and health workers at the Hospital Universitario Oswaldo Cruz with a PCR-confirmed COVID-19 infection between March and June 2020 were considered for the study and were interviewed by telephone at least 2-months after the acute phase of the disease. These patients were identified by the hospital epidemiological surveillance department. A semi-structured questionnaire was used containing sociodemographic and clinical data and the ID-Migraine.

**Results:**

A total of 288 patients was interviewed; 53.1% were male; with a median age of 49.9 (41.5–60.5) years; 91.7% presented some neurological symptom; 22.2% reported some neurological symptom as the symptom that troubled them most during COVID-19. Neurological symptoms were: ageusia (69.8%), headache (69.1%), anosmia (67%), myalgia (44.4%), drowsiness (37.2%), agitation (20.8%); mental confusion (14.9%), syncope (4.9%) and epileptic seizures (2.8%). Females, those who presented with fever, sore throat, anosmia/ageusia and myalgia also presented significantly more with headache (logistic regression). The most frequent headache phenotype was a non-migraine phenotype, was of severe intensity and differed from previous headaches. This persisted for more than 30 days in 18% and for more than 90 days in 10% of patients. Thirteen percent of those with anosmia and 11% with ageusia continued with these complaints after more than 90 days of the acute phase of the disease. Aged over 50 years, agitation and epileptic seizures were significantly associated with mental confusion (logistic regression).

**Conclusion:**

Headache is frequent in COVID-19, is associated with other symptoms such as fever, sore throat, anosmia, ageusia, and myalgia, and may persist beyond the acute phase of the disease.

**Supplementary Information:**

The online version contains supplementary material available at 10.1186/s10194-021-01367-8.

## Background

During the COVID-19 pandemic, neurological symptoms and neurological diseases have been described, thereby reinforcing the possible involvement of both the central nervous system and the peripheral nervous system [1–5]. This involvement may occur during viral invasion, due to the systemic inflammatory process, hypoxia, vascular complications and postviral immune-mediated complications [6].

Among the neurological diseases, there are reports of cerebrovascular diseases, encephalopathies, encephalitis, meningitis, myelitis, acute disseminated encephalomyelitis, Guillain-Barré syndrome, mononeuropathies and myopathies [1–5]. However, neurological diseases are more frequent in the most severe spectrum of the disease [7], and appear to be rare in terms of population. A population study in Singapore reported a 0.08% incidence of neurological diseases among those infected with SARS-CoV 2. This incidence does not include neurological symptoms, and anosmia and ageusia were not considered neurological diseases in this study [5].

On the other hand, neurological symptoms are much more common than neurological diseases and occur in 8 to 91% of hospitalized patients [2, 4, 8–10]. One study, conducted in two Spanish hospitals, reported that 2.5% of the 841 inpatients had sought hospital care due to neurological symptoms [9]. The most frequently reported neurological symptoms are headache, anosmia, ageusia, myalgia, mental confusion and an altered level of consciousness [1–4, 7–10]. These symptoms generally occur during the first days of COVID-19 symptoms [9–11] and headache, anosmia and ageusia are more likely to occur together [12–15]. In certain patients, some of these symptoms may persist beyond the acute phase of the disease [16]. Little is known regarding the repercussions of neurological symptoms for patients and how these symptoms are related to one another.

This study has aimed to determine whether there is an association between the neurological symptoms in patients with COVID-19, and also to characterize the headache presented by these patients.

## Method

This was a cross-sectional study with a retrospective assessment conducted at the Hospital Universitario Oswaldo Cruz. This is a reference hospital for infectious diseases in the State of Pernambuco, Northeastern Brazil.

All adult inpatients and healthcare workers registered between March and June 2020 by the hospital’s Epidemiological Surveillance Department as being suspected COVID-19 cases were identified, together with their telephone numbers. The inpatients had been referred from secondary hospitals that did not admit COVID-19 patients, and from emergency services in other hospitals.

All the patients aged over 18 years, diagnosed with COVID-19 confirmed by reverse transcription polymerase chain reaction (RT-PCR) technique, from material collected by nasal and oropharynx swab, were included in the study. No patient was diagnosed solely based on clinical symptoms. Patients who had any cognitive impairment that prevented the interview from being conducted, and those who died before the interview, were excluded.

Patients were tested by different kits of RT-PCR, according to the availability of the public health system at the time of the assessment: Molecular Kit SARS-Cov-2(E/P1) (Bio-Manguinhos, Fiocruz, Rio de Janeiro, Brazil), BIOMOL Kit OneStep/COVID-19 (IBMP, Paraná, Brazil), AllplexTM 2019-nCov Assay (Seegene Inc., Minas Gerais, Brazil), and 2019-nCov CDC Assay (IDT Inc., Iowa, USA), respectively with a detection limit of 50, 6, 100, and 8 copies per reaction. The tested specificity of all the kits was > 99%.

### Data collection

Patients were contacted by telephone at least 2-months after the acute phase of COVID-19, and were interviewed with regard to the symptoms presented during this phase. The onset of the acute phase was considered as the onset of the first symptom. Interviews were conducted from June to November 2020 by specifically trained resident neurology doctors and one medical student. Patients who presented with some degree of cognitive impairment or altered consciousness that prevented verbal communication were excluded.

Interviews were conducted using a semi-structured questionnaire. The first part of the questionnaire contained sociodemographic information, data regarding previously presented diseases (comorbidities) and a list of general symptoms and neurological symptoms, such as headache, myalgia (“Pain in the body (muscles)”), changes in the perception of smell (“Decreased smelling ability” and “I couldn’t smell things”) and of taste (“Decreased taste of food” and “I couldn’t taste the food”), agitation (“Irritation/agitation”), a loss of consciousness (“fainting”), mental confusion, drowsiness (“Drowsiness (unable to stay awake)”) and epileptic seizures. Each of these symptoms had dichotomous yes/no responses. Patients were also asked which symptom had troubled them the most during COVID-19.

The second part of the questionnaire was answered only by those who reported having headache, changes in smell and/or taste, plus details of the characteristics of these symptoms.

To classify the phenotype of the headache presented during COVID-19, we used the Portuguese version of ID-Migraine. Those who obtained a score greater than or equal to 2 were considered as presenting “phenotypic migraine-like features” (a sensitivity of 92% and a positive predictive value of 93% for migraine diagnosis) [17].

In order to determine the time interval between the symptomatic phase of COVID-19 and the interview, the specimen collection date for the RT-PCR test and the date of the interview were used as a reference.

All patients provided an oral consent, and the research was approved by the National Research Ethics Commission in Brazil (CONEP; CAAE: 30479220.8.0000.5192; Report Number: 4.082.904).

### Availability of data and materials

The data that support the findings of this study are available from the corresponding author upon reasonable request.

### Data analysis

The statistical analyses were performed using SPSS 21.0 (IBM Corporation, Armonk, NY, USA).

Quantitative data were presented as medians and interquartile range (percentiles 25–75) since all distribution was non-normal according to the Kolmogorov-Smirnov test.

The percentage distribution of the categorical variables was compared between the groups by means of the Chi-square test or Fisher’s exact test. Numerical variables were compared using the Mann-Whitney test.

Considering the existence of subsets of variables correlated with one another, which indicated aspects of the same dimension, we performed an exploratory factorial analysis with the principal component method and oblique rotation (direct oblimin). The Kaiser-Meyer-Olkin (KMO) measure and the Bartlett’s test of sphericity indicated the adequacy of the model. Variables with factorial loadings over 0.4 were grouped according to their scores.

We also performed a logistic regression analysis to identify associations between the variables that presented the highest load among the encountered factors and the other sociodemographic and clinical factors. We previously defined using the pairwise deletion method in the analysis to address any missing data, but this proved to be unnecessary since we were able to collect data from all patients for the variables included in the regression models. We adjusted for possible confounders using these multivariate regression models. Variables were selected for the logistic regression models according to a *p*-value < 0.10 in the univariate analysis. The stepwise method was used and we first included those variables with the highest values of the Qui-square test. Variables were maintained in the final model according to the stability of the model by comparing the standard error variation in order to avoid collinearity. Results are presented as bias-corrected odds ratios (OR) and 95% confidence intervals (CI) based on the analyses of 1000 computer-generated bootstrap samples.

Post-hoc analysis using the Bonferroni method was used to avoid the problem of multiple comparisons.

All tests were leveled by a 0.05 significance.

## Results

**Fig. 1:**
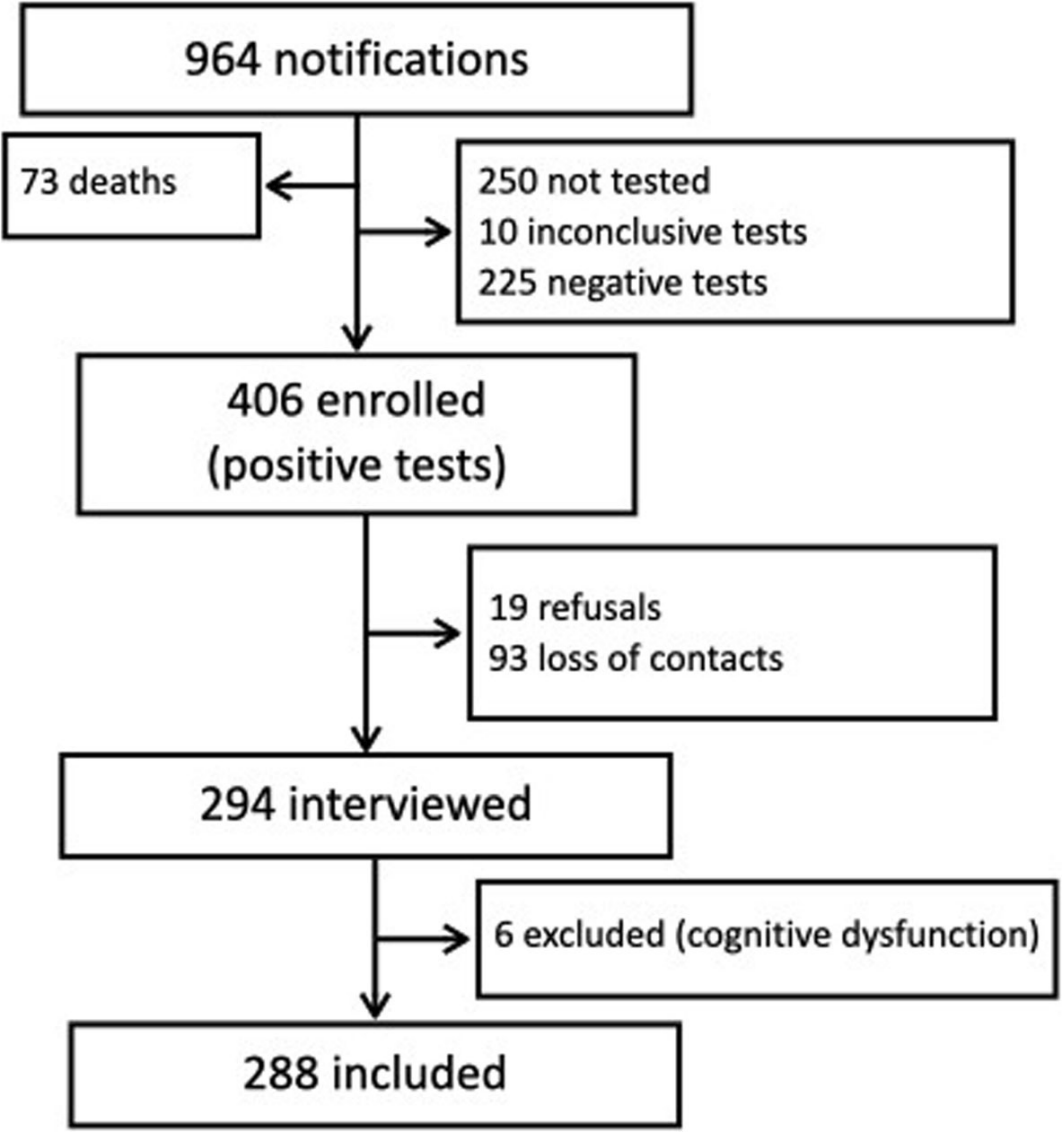
Study flowchart

During the assessed period, 964 patients were reported as being possible cases of COVID-19, of whom, 406 patients had a positive RT-PCR test for SARS-CoV 2 and were therefore eligible for the research. There were 112/406 losses (27.6%) and six patients were excluded (Fig. 1).

Two hundred and eighty-eight patients were interviewed. The median time between the symptomatic phase and the interview was 111.5 (86–141.5) days. The median age was 49.9 (41.5–60.5) years and 153/288 (53.1%) of the respondents were male.

Two hundred and twelve (73.6%) patients reported some comorbidity: arterial hypertension (121/288; 42%), overweight (114/288; 39.6%), diabetes mellitus (78/288; 27.1%), asthma (29/288; 10.1%); cardiac arrhythmia (17/288; 6%), kidney disease (12/288; 4%), heart failure (10/288; 3.5%), myocardial infarction (8/288; 2.8%), epilepsy (8/288; 2.8%), liver disease (3/288; 1%) and Parkinson’s disease (1/288; 0.4%).

Table 1 demonstrates the symptoms presented during COVID-19. The most frequent neurological symptoms were ageusia, headache, anosmia and myalgia.

**Table 1:** Symptoms presented by patients with COVID-19

Symptoms	Total	
	(***n*** = 288)	CI95%
**Difficulty in breathing (Dyspnoea)**	223 (77.4%)	72.2–82.1
**Fever**	199 (69.1%)	63.4–74.4
**Cough**	196 (68.1%)	62.3–73.4
**Fatigue**	196 (68.1%)	62.3–73.4
**Drop in oxygen saturation**	165 (57.3%)	51.4–63.1
**Chills**	119 (41.3%)	35.6–47.3
**Diarrheia**	111 (38.5%)	32.9–44.4
**Joint pains**	103 (35.8%)	30.2–41.6
**Sore throat**	103 (35.8%)	30.2–41.6
**Stuffy nose**	99 (34.4%)	28.9–40.2
**Tight chest**	99 (34.4%)	28.9–40.2
**Nausea**	94 (32.6%)	27.3–38.4
**Abdominal pain**	70 (24.3%)	19.5–29.7
**Vomiting**	67 (23.3%)	18.5–28.6
**Cyanosis**	25 (8.7%)	5.7–12.6
**NEUROLOGICAL SYMPTOMS**		
**Any neurological symptom**	264 (91.7%)	87.9–94.6
**Hypogeusia/ageusia**	201 (69.8%)	64.1–75
**Headache**	199 (69.1%)	63.4–74.4
**Hyposmia/anosmia**	193 (67.0%)	61.3–72.4
**Myalgia**	128 (44.4%)	38.6–50.4
**Drowsiness**	107 (37.2%)	31.6–43
**Agitation**	60 (20.8%)	16.3–26
**Mental confusion**	43 (14.9%)	11–19.6
**Syncope**	14 (4.9%)	2.7–8
**Epileptic seizure**	8 (2.8%)	1.2–5.4

One hundred and sixty patients reported some respiratory symptom, 91.7% (264/288) reported some neurological symptom, and 64/288 (22.2%) reported some neurological symptom as being the symptom that troubled them the most during COVID-19. Fifty-eight patients (20.1%; 58/288) reported some painful complaint as being that which troubled them the most during the disease. The complaint that troubled patients the most was headache for 27/288 (9.4%), ageusia for 10/288 (3.5%), anosmia for 9/288 (3.1%) and myalgia for 8/288 (2.8%) of the interviewees.

Anosmia was the first symptom of COVID-19 in 56/193 (29%) and began at the same time as the other symptoms in 63/193 (32.6%) of those who reported this complaint. Twenty-five people still reported anosmia at the time of the interview (more than three months after the acute phase of the disease), and of these, 20 reported that it was improving, and five stated that it remained unchanged. The median duration of anosmia among those who no longer presented this symptom was 9 (7.5–15) days, and the maximum duration was 60 days.

Ageusia was the first symptom of COVID-19 in 55/201 (27.4%) and began at the same time as the other symptoms in 74/201 (36.8%) of those who presented this complaint. Twenty-two patients still reported having ageusia at the time of the interview (more than three months after the acute phase of the disease), and of these, 18 reported that it was improving and four patients stated that it remained unchanged. The median duration of ageusia among those who no longer had this symptom was 10 (7–16) days and the maximum duration was 60 days.

## Headache

Headache was the first reported symptom in 84/199 (42.2%) and began along with the other symptoms of COVID-19 in 75/199 (37.7%).

Of the 199 patients who reported headache during the symptomatic phase of COVID-19, 27 (13.6%) continued to present with this symptom. The median time between the symptomatic phase of these patients and the interview was 103 (79–170) days. Twenty patients reported that the headache was improving, although six stated that it remained unchanged. In 20/199 (10.1%), the headache had lasted for more than three months.

The median duration of headache among the 172 who no longer had this symptom was 7 (4.5–12.5) days. In nine patients, the headache had lasted for a period of 30 to 60 days.

The median intensity of headache, using a scale from 1 to 10, was 8 (5.5–9.5). Headache was associated with nausea in 85/199 (42.7%), photophobia in 97/199 (48.7%), limiting daily activities in 105/199 (52.8%), and presented phenotypic migraine-like features (ID-Migraine greater than or equal to 2) in 98/199 (49.3%, 95% CI 42.1–56.4) of cases. Ninety-two patients (46.2%; 92/199) who presented with headache reported having a previous headache and, of these, 78.3% (72/92) assessed the COVID-19-associated headache as being different from any previous headaches.

No difference was recorded between those who did or did not have anosmia in the medians of intensity [8 (6–9) vs. 8 (5–8) points, *p* = 0.232] or duration [7 (4.5–14.5) vs. 6 (3.5–8) days; *p* = 0.071) of headache. There was also no association between having ageusia and the medians of the duration [7 (4.5–14) vs. 6 (3–10) days, *p* = 0.327] or intensity [8 (6.5–9) vs. 7 (5.5–8), *p* = 0.374] of headache.

### Association between symptoms

**Fig. 2:**
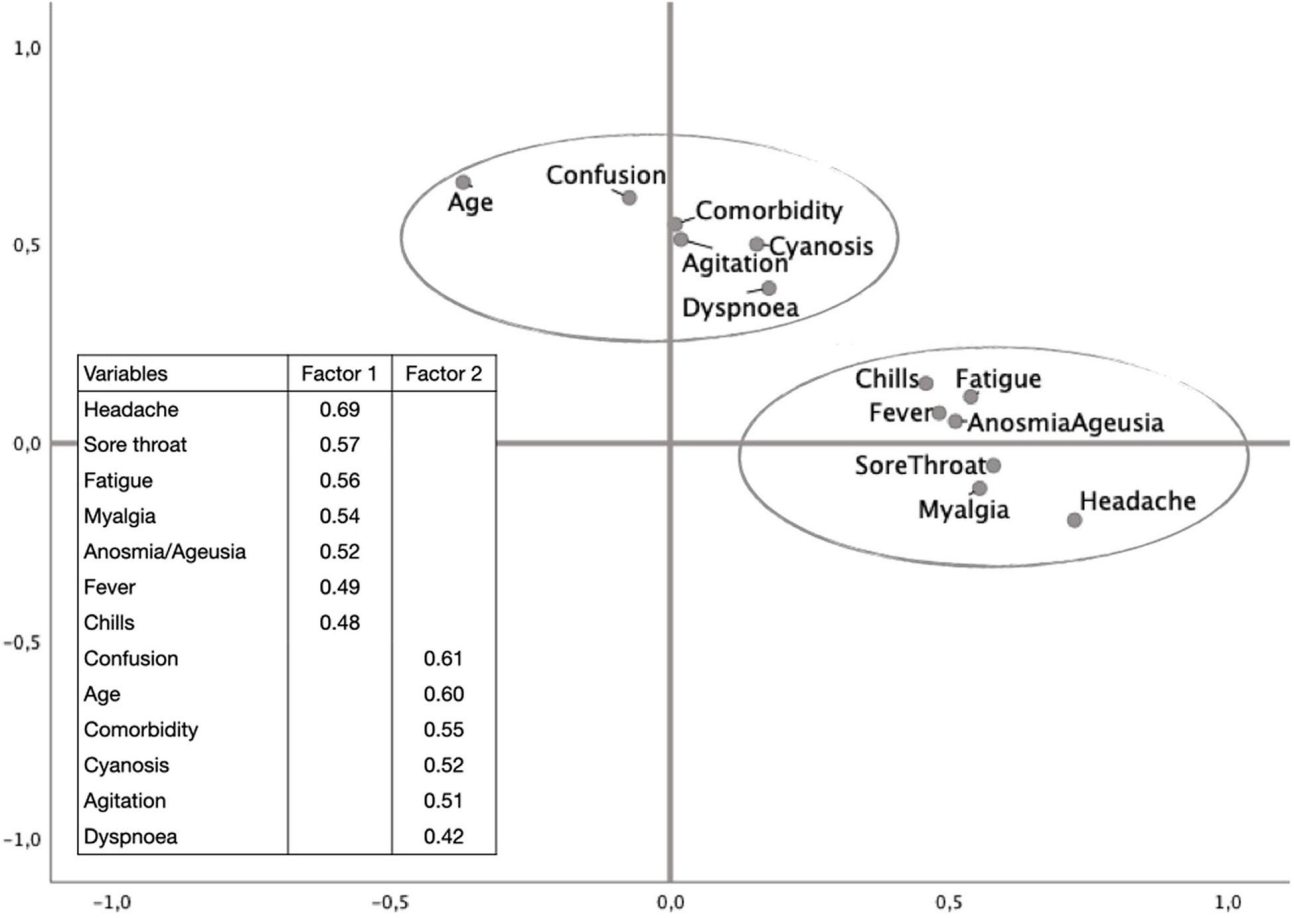
Rotated factor loadings plot of each variables included in the final model (KMO measure = 0.71, Bartlett’s test of sphericity < 0.001; Total variance explained = 31.7%; Factor correlation = 0.16)

All complaints were included in the factor analysis and those with a factor load above 0.4 were maintained. The KMO measure was 0.71, indicating that the sample was adequate for a significance of less than 0.001 with the Bartlett’s test of sphericity. The final model enabled the observation of two independent factors as indicated by the factor correlation of 0.16 (Fig. 2). The first factor included headache (highest load), sore throat, fatigue, myalgia, anosmia/ageusia, fever and chills. Taken together, they would seem to indicate the symptoms that predominate in the early stage of the disease. The second factor included mental confusion (highest load), aged over 50 years, comorbidity, cyanosis, agitation and dyspnea, which together, would seem to indicate a more severe stage of the disease.

Taking into account the symptoms with the highest factor load, two multivariate analyzes were performed. Table 2 presents the association between the sociodemographic and clinical characteristics with headache. After controlling for confounding variables, females, those presenting with fever, sore throat, anosmia/ageusia and myalgia remained significantly associated with headache.

**Table 2:** Estimated risk for headache according to patient sociodemographic and clinical characteristics

Characteristics	Headache ***N*** = 199	Without headache ***N*** = 89	Total N = 288	***p*** value	Raw OR (95% CI)	Adjusted OR (95% CI)^a^
**Female sex, n (%)**	111 (55.8)	24 (27)	135 (49.9)	< 0.01	3.42 (1.98–5.90)	3.13 (1.64—5.97)
**Aged > 50 years, n (%)**	91 (45.7)	53 (59.6)	144 (50)	0.03	0.57 (0.35—0.95)	0.97 (0.95—1.01)
**Comorbidity, n (%)**	151 (75.9)	61 (68.5)	212 (73.6)	0.19	1.44 (0.83—2.51)	–
FLU SYMPTOMS:						
**Fever, n (%)**	157 (78.9)	42 (47.2)	199 (69.1)	< 0.01	4.18 (2.44—7.16)	3.99 (2.10—7.63)
**Chills, n(%)**	95 (47.7)	24 (27)	119 (41.3)	< 0.01	2.47 (1.44—2.47)	–
**Cough, n(%)**	141 (10.9)	55 (61.8)	196 (68.1)	0.13	1.50 (0.89—2.54)	–
**Sore throat, n(%)**	92 (46.2)	11 (12.4)	103 (35.8)	< 0.01	6.10 (3.06—12.16)	3.69 (1.71—7.93)
**Dyspnoea, n (%)**	159 (79.9)	64 (71.9)	223 (77.4)	0.13	1.55 (0.87—2.77)	–
**Chest pain, n (%)**	83 (41.7)	16 (18)	99 (34.4)	< 0.01	3.27 (1.77—6.01)	–
**Low oxygenation, n (%)**	111 (55.8)	54 (60.7)	165 (57.3)	0.44	0.82 (0.49—1.36)	–
**Cyanosis, n (%)**	20 (10.1)	5 (5.6)	25 (8.7)	0.22	1.88 (0.68—5.17)	–
**Coryza, n (%)**	80 (40.2)	19 (21.3)	99 (34.4)	< 0.01	2.48 (1.39—4.43)	–
**Joint pain, n (%)**	87 (43.7)	16 (18)	103 (35.8)	< 0.01	3.54 (1.93—6.52)	–
**Diarrhea, n (%)**	91 (45.7)	20 (22.5)	111 (38.5)	< 0.01	2.91 (1.64—5.14)	–
**Nausea, n (%)**	80 (40.2)	14 (15.7)	94 (32.6)	< 0.01	3.60 (1.91—6.81)	–
**Vomiting, n (%)**	56 (28.1)	11 (12.4)	67 (23.3)	< 0.01	2.78 (1.38—5.61)	–
**Abdominal pain, n (%)**	61 (30.7)	9 (10.1)	70 (24.3)	< 0.01	3.93 (1.85—8.34)	–
**Flu, n (%)**	198 (99.5)	78 (87.6)	276 (95.8)	< 0.01	–	–
**SARS, n (%)**	199 (100)	83 (93.3)	282 (97.9)	< 0.01	–	–
NEUROLOGICAL SYMPTOMS:						
**Anosmia/Ageusia, n (%)**	160 (80.4)	48 (53.9)	208 (72.2)	< 0.01	3.50 (2.03—6.04)	1.94 (1.01—3.73)
**Myalgia, n (%)**	107 (53.8)	21 (23.6)	28 (44.4)	< 0.01	3.77 (2.15—6.61)	2.40 (1.26—4.59)
**Fatigue, n (%)**	150 (75.4)	46 (51.7)	196 (68.1)	< 0.01	2.86 (1.69—4.84)	1.56 (0.82—2.99)
**Somnolence, n (%)**	80 (40.2)	27 (30.3)	107 (37.2)	0.11	1.54 (0.91—2.63)	–
**Confusion, n (%)**	26 (13.1)	17 (19.1)	43 (14.9)	0.18	0.64 (0.33—1.24)	–
**Agitation, n (%)**	41 (20.6)	19 (21.3)	60 (20.8)	0.89	0.96 (0.52—1.76)	–
**Syncope, n (%)**	7 (3,5)	7 (7.9)	14 (4.9)	0.11	0.43 (0.15—1.26)	–
**Seizure, n (%)**	7 (3,5)	1 (1.1)	8 (2.8)	0.25	3.21 (0.39—26.47)	–

Variables considered to the model: sex, age, fever, chills, sore throat, chest pain, coryza, joint pain, diarrhea, nausea, vomiting, abdominal pain, flu, SARS, anosmia/ageusia, myalgia, fatigue

^a^Final Model: myalgia, anosmia/ageusia, sex, sore throat, fever, fatigue, and age

Table 3 presents the association between the sociodemographic and clinical characteristics with mental confusion. After controlling for confounding variables, aged over 50 years, agitation and epileptic seizures remained significantly associated with mental confusion.

**Table 3:** Estimated risk for mental confusion according to patient sociodemographic and clinical characteristics

Characteristics	Confusion ***N*** = 43	Without confusion ***N*** = 245	Total N = 288	p value	Raw OR (95% CI)	Adjusted OR (95% CI)^a^
**Female sex, n (%)**	18 (41.9)	117 (47.8)	135 (46.9)	0.48	0.78 (0.41–1.52)	–
**Aged > 50 years, n (%)**	32 (74.4)	112 (45.7)	144 (50)	< 0.01	3.46 (1.67—7.17)	3.76 (1.54—9.19)
**Comorbidity, n (%)**	40 (93)	172 (70.2)	212 (73.6)	< 0.01	5.66 (1.70—18.88)	3.13 (0.84—11.68)
FLU SYMPTOMS:						
**Fever, n (%)**	32 (74.4)	167 (68.2)	199 (69.1)	0.41	1.36 (0.65—2.84)	–
**Chills, n(%)**	21 (48.8)	98 (40)	119 (41.3)	0.28	1.43 (0.75—2.74)	–
**Cough, n(%)**	32 (74.4)	164 (67)	196 (68.1)	0.33	1.44 (0.69—3.00)	–
**Sore throat, n(%)**	17 (39.5)	86 (35.1)	103 (35.8)	0.58	1.21 (0.62—2.35)	–
**Dyspnoea, n (%)**	38 (88.4)	185 (75.5)	223 (77.4)	0.06	2.47 (0.93—6.55)	–
**Chest pain, n (%)**	18 (42)	88 (33.1)	99 (34.4)	0.26	1.46 (0.75—2.83)	–
**Low oxygenation, n (%)**	33 (76.7)	132 (54)	165 (57.3)	< 0.01	2.83 (1.33—5.98)	2.11 (0.91—4.92)
**Cyanosis, n (%)**	9 (21)	16 (6.5)	25 (8.7)	< 0.01	3.79 (1.55—9.25)	1.29 (0.45—3.72)
**Coryza, n (%)**	17 (39.5)	82 (33.5)	99 (34.4)	0.44	1.33 (0.67—2.53)	–
**Joint pain, n (%)**	21 (48.8)	82 (33.5)	103 (35.8)	0.05	1.90 (0.99—3.65)	–
**Diarrhea, n (%)**	16 (37.2)	95 (38.8)	111 (38.5)	0.85	0.94 (0.48—1.83)	–
**Nausea, n (%)**	14 (32.6)	80 (32.7)	94 (32.6)	0.99	0.99 (0.50—1.99)	–
**Vomiting, n (%)**	12 (28)	55 (22.4)	67 (23.3)	0.44	1.34 (0.64—2.78)	–
**Abdominal pain, n (%)**	19 (44.2)	51 (21)	70 (24.3)	< 0.01	3.01 (1.53—5.92)	–
**Flu, n (%)**	42 (97.7)	234 (95.5)	276 (95.8)	0.51	–	–
**SARS, n (%)**	43 (100)	239 (97.6)	282 (98)	0.30	–	–
NEUROLOGICAL SYMPTOMS:						
**Headache, n (%)**	26 (60.5)	173 (70.6)	199 (69.1)	0.18	0.64 (0.33—1.24)	–
**Anosmia/Ageusia, n (%)**	33 (76.7)	175 (71.4)	208 (72.2)	0.47	1.32 (0.62—2.82)	–
**Myalgia, n (%)**	21 (48.8)	107 (43.7)	128 (44.4)	0.53	1.23 (0.64—2.36)	–
**Fatigue, n (%)**	32 (74.4)	164 (67)	196 (68.1)	0.33	1.44 (0.69—3.00)	–
**Somnolence, n (%)**	22 (51.2)	85 (34.7)	107 (37.2)	0.04	1.97 (1.03—3.79)	1.33 (0.62—2.86)
**Agitation, n (%)**	18 (42)	42 (17.1)	60 (20.8)	< 0.01	3.48 (1.74—6.95)	2.76 (1.29—5.94)
**Syncope, n (%)**	4 (9.3)	10 (4.1)	14 (5)	0.14	2.41 (0.72—8.07)	–
**Seizure, n (%)**	5 (11.6)	3 (1.2)	8 (2.8)	< 0.01	10.61 (2.44—46.24)	27.2 (4.52—164.6)

Variables considered to the model: age, comorbidity, dyspnoea, low oxygenation, cyanosis, joint pain, abdominal pain, somnolence, agitation, Seizure

^a^Final Model: seizure, agitation, age, comorbidity, cyanosis, low oxygenation, and somnolence

## Discussion

We observed a high frequency of neurological symptoms in patients with COVID-19, which, among all the symptoms, were the complaints that most troubled 22% of patients. The most frequent neurological symptoms were ageusia, headache, anosmia and myalgia, which is in accordance with the literature [2–4, 7–9].

Our attention is drawn to the fact that the painful symptoms were extremely frequent and that they were among those that most troubled patients. In addition to headache and myalgia, joint pain, sore throat and abdominal pain were also frequently reported. Painful symptoms are rarely studied and little attention is paid to them by doctors in COVID-19, especially when faced with respiratory complaints, which have the greatest potential for causing complications. Our results reinforce the importance of actively researching pain and treating it appropriately.

We observed a high prevalence of headache, which was one of the symptoms that most troubled our interviewees, and thereby interfered with their daily activities. In another study, which only included patients who presented with headache, this was the symptom that most troubled 18.9% of patients [18]. In another study carried out in Spain that included both in-patients and out-patients, headache was the symptom that most bothered 15.6% of patients [19]. A recent systematic review found a prevalence of headache in the acute phase of COVID-19 of 47.1% (95%CI: 35.8–58.6%) of patients, which is similar to our findings [20].

In our study, headache was more frequent in females and in patients with fever. While females have also presented a higher risk of headache in the acute phase of COVID-19 in other studies [8, 12, 21, 22], this was not the case in all studies [7, 13, 14, 23–26].

Fever is believed to be an important factor in the development of headache associated with systemic viral infections [18]. Despite this, only a very few studies have reported an association between headache and fever in the acute phase of COVID-19 [12, 22, 27] and most studies have not reported this association [14, 21, 23, 25]. Indeed, thus far, the possible association between headache and fever has not been the primary research question of any study. However, there is a biological plausibility in an association between fever and headache in COVID-19. While some studies have reported an association between the occurrence of headache and pro-inflammatory substances such as NLRP3, HMGB1, and interleukin 6, [2, 28] this has not been the case with all authors [21, 26, 29]. Moreover, a comparison between these studies is difficult because the levels of these substances were measured at different times.

When present, headache was generally the first to appear among all the COVID-19 symptoms, lasted for at least seven days, had a non-migraine phenotype, was of strong intensity and was different from previous headaches. The headache persisted for more than 30 days in 18% of those who reported headache and lasted for more than 3 months after the acute phase of COVID-19 in 10%. Together, these data demonstrate that headache is not a minor problem, but a significant symptom of COVID-19.

The predominance of characteristics such as presenting early onset, [12–14, 18, 24, 25, 30] of strong intensity, [13, 14, 24, 30] and being different from previous headaches [13, 14, 24, 25], have also been reported by other studies. The predominant phenotype of headache in COVID-19 (migraine or tension-type headache phenotype) is, however, controversial in the literature [7, 13, 18, 22, 31].

Our results corroborate with the few studies that have assessed the temporal behavior of headache and have reported that it may persist beyond the acute phase of the disease [7, 18–21, 23, 32, 33]. Persistent post-COVID-19 headache may be the result of multiple mechanisms such as direct neuro-invasion with damage along the neuronal pathway, the indirect effects of hypoxia, coagulopathy, and cytokine storm [34, 35]. Monitoring patients after the acute phase of the disease, even by telephone, may help to identify this headache so that it may be treated early, thereby reducing its impact.

We observed a high prevalence of anosmia and ageusia, which generally occurred at the onset of symptoms. The prevalence in our study is within the range described in the literature, although it is higher than most studies [36, 37]. The fact that patients were less severe [7, 37] and that data were obtained by direct patient interviews may have contributed to such a high prevalence.

By exploring the symptoms together, we encountered the presence of two groups of symptoms. The first includes headache, anosmia, ageusia and myalgia. The second includes mental confusion and agitation. Some flu-like symptoms were also grouped together with these neurological symptoms. As far as we are aware, this is the first study to demonstrate these two groupings in a theoretical model. Factor analysis, as a way of exploring a large set of symptoms, enabled us to observe an appropriate model for our sample, in which these groupings of symptoms seem to demonstrate common characteristics, in addition to appearing statistically reasonable to be able to assume their independence. These groupings of symptoms observed in our sample may be related to different stages of the disease, to common pathophysiological processes or to the severity of the patient [38–40].

In addition to being grouped in the factor analysis, headache was also associated with anosmia/ageusia and myalgia in the logistic regression model. Associations between ageusia and anosmia, [23, 41] between headache and myalgia, [12, 31] between headache and anosmia [12, 13, 21] and between headache and ageusia [13, 21] have all been previously described. Headache, anosmia and ageusia usually occur at the beginning of the symptomatic period of COVID-19 [12, 13, 18, 25, 30, 42] and are more frequent in less severe cases [1, 7, 9, 12, 21, 25, 31, 37]. The identification of micro-bleeds in the olfactory bulb in patients with persistent headache and anosmia reinforces the hypothesis of a direct viral injury or vascular injury in the genesis of these two symptoms [43].

Mental confusion was associated with agitation and epileptic seizures. These symptoms occur more frequently in cases of greater severity, [1, 9, 44, 45] and therefore have a higher frequency in a hospital series [1, 10, 44]. The most severe phase of the disease generally occurs from the second week of the disease [39]. The systemic inflammatory process, hypoxia, and drug use may be significant factors for the occurrence of these symptoms, [38–40] which may also be associated with the development of an encephalopathic condition [40].

Our study has some limitations. The patients included were from a single center. This may limit the generalization of the study concerning milder cases. The epidemiological profile of health workers differs from those who presented to the emergency department and were hospitalized. Although the great majority of our patients were hospitalized, the patient list, provided by the hospital, does not indicate how many were inpatients and how many were health professionals who were treated as outpatients. Only patients who had been discharged and who were able to answer the questions were included in the study. This may have led to an underrepresentation of the more severe cases. The interview was conducted at least 2 months after the symptoms, which increases the chance of memory bias. The time interval between the acute phase of COVID-19 and the interview varied among patients. The semi-structured questionnaire used was not validated and the quality, duration, and localization of the headache were not included in the questionnaire. The factorial analysis performed has an exploratory character and only enables interpretations within the studied sample and, although the obtained results present biological plausibility, they need to be corroborated by other studies. As a sample size calculation was not performed, we cannot rule out the possibility that small differences might not have been identified.

Losses accounted for 27.6%. We cannot rule out the possibility that this may have interfered with the internal validity of the study. However, the frequency of neurological symptoms we observed is in agreement with that reported in the literature.

The most suitable study design to assess the duration of symptoms is the cohort study. Therefore, we must interpret this data carefully. However, since we know the dates on which the RT-PCR tests were performed (acute phase of the disease) and the interview dates, this data was used to estimate this duration.

A total of 250 patients had no RT-PCR results. The hospital protocol established that all inpatients or healthcare professionals who presented with symptoms suggestive of COVID-19 should be tested. However, as these patients were infected in the first wave of COVID-19 in Brazil and our healthcare system was in disarray, not all of the collected tests were processed. These tests were not processed at our hospital. Thus, no form of selection process was adopted in order to choose which patients should perform the RT-PCR. We therefore decided that only patients with positive RT-PCR tests should remain in the study so as to reduce misclassification.

Our study has some strengths. In the period chosen for the selection of patients, those who had been admitted to the Hospital Universitario Oswaldo Cruz represented an expressive number of patients who needed hospitalization in the state of Pernambuco. Most of the interviews were conducted by trained doctors, experienced in addressing neurological symptoms. As the data were obtained by interview, the chance of information loss was decreased. Moreover, all patients included in the study had a positive RT-PCR test for SARS-CoV 2, thereby decreasing the chance of misclassification.

## Conclusion

Neurological symptoms are frequent and significant in the acute phase of COVID-19. The most frequent neurological symptoms were ageusia, headache, anosmia and myalgia. Headache, anosmia and ageusia may persist beyond the acute phase of the disease. Headache was associated with females, fever, sore throat, ageusia/anosmia and myalgia. Mental confusion was associated with older age, agitation and epileptic seizures.

## Supplementary Information

**Additional file 1.**

## Data Availability

The datasets used and/or analysed during the current study are available from the corresponding author upon reasonable request.
