# Supplementary material for: Headache, anosmia, ageusia and other neurological symptoms in COVID-19: a cross-sectional study
Source: J Headache Pain. 2022 Jan 3;23(1):2. doi: 10.1186/s10194-021-01367-8 (PMC8721484; doi:10.1186/s10194-021-01367-8)
Supplement: Supplementary file 1 — Additional file 1. [file 10194_2021_1367_MOESM1_ESM.docx]

PHONE INTERVIEW

Who did the interview: ____________________ Date: _________________________

Name: ____________________________________________________________

 Doesn't want to answer

Not found:  1○ Attempt  2○ Attempt  3○ Attempt

We are researching the symptoms related to virus that give flu symptoms, such as the coronavirus and influenza virus. These questions are about this subject.

1 - What did you feel when you had the flu?

Fever (or feverish feeling)  Chills  Cough  Sore throat

 Difficulty breathing  Tightness in the chest  Decrease in oxygenation

 Purple lips  Coryza (clogged/runny nose)

 Decreased smelling ability  I couldn't smell things [QUESTION 3]

 Decreased taste of food  I couldn't taste the food [QUESTION 4]

 Diarrhea  Nausea  Vomiting  Stomach (abdominal) pain

 Pain in the body (muscles?)  Pain in the joints (joints)

 Headache [QUESTION 5]

 Fatigue  Drowsiness (unable to stay awake)

 Mental Confusion  Irritation/Agitation

 Fainting  Convulsion

 Others______________________________________________________

2 – Of all these symptoms, which one bothered the most?

3 - If you checked Decrease in smell/ I couldn't smell things, ask:

3.1 Regarding the other flu symptoms, the change in smell appeared:

 Before  At the same time  After

3.2 The smell change has started:

 Suddenly, all of a sudden  It was appearing little by little

3.3 Is there still a change in the smell?

 Yes. So it is:  Getting better  Equal  Getting worse

 No. So how many days did it last? ______________

4 - If you checked Decreased taste of food/ I couldn't taste the food, ask:

4.1 Regarding the other flu symptoms, the change in taste appeared:

 Before  At the same time  After

4.2 The taste change has started:

 Suddenly, all of a sudden  It was appearing little by little

4.3 Do you still have a change in taste?

 Yes. So she is:  Getting better  Equal  Getting worse

 No. So how many days did it last? ______________

5 - If you have a headache, ask:

The following questions are about the headache you felt from the flu

5.1 Regarding the other flu symptoms, the headache started:

 Before  At the same time  After

5.2 From zero to ten, what would you rate your headache, with zero being "no pain" and 10 "worst pain in life": _____

5.3 Have any of the following symptoms associated with headache occurred

 Did you ever feel nauseous when you had headache pain?

 Did the light trouble you (much more than then when there is no headache)?

 Did your headache ever limit your ability to work, study or do something you needed to?

5.4 Do you still have a headache?

 Yes. So she is:  Getting better  Equal  Getting worse

 No. So how many days did it last? ______________

5.5 Did you have a headache before this flu?

 Yes. So, the flu headache is:  Equal  Different

 No

6 - Do you have any illness or are you undergoing treatment for any illness?

 High Blood Pressure  Diabetes  High Cholesterol  Overweight

 Heart infarction  Arrhythmia  Overgrown heart  Stroke

 Kidney disease  Cirrhosis  Asthma/bronchitis

 Neurological disease:  Epilepsy  Alzheimer  Parkinson

 Others: ______________________________________________________________

Do you use any medication: __________________________________________________________________________________________________________________________________________________________________________________________________________________
